# EIT comparison of airway pressure release ventilation and conventional ventilation

**DOI:** 10.1186/cc13481

**Published:** 2014-03-17

**Authors:** S Jog, S Sable, D Patel, P Tambur

**Affiliations:** 1 Deenanath Mangeshkar Hospital and Research Center, Pune, India

## Introduction

The aim was to study EIT as a monitoring tool for tidal ventilation (TV) redistribution following switching patients from volume-controlled ventilation (VCV) to airway pressure release ventilation (APRV) in patients with severe ARDS.

## Methods

Six patients with severe ARDS having Pplat .30 cm were included in the study. Patients ventilated with the ARDSnet strategy were subjected to EIT analysis. Regional TV distribution was monitored by an EIT system (PulmoVista 500R; Drager Medical GmbH, Lubeck, Germany), dividing the lung field into four same-size regions of interest(ROIs): ventral right (ROI 1) and left (ROI 2) and dorsal right (ROI 3) and left (ROI 4). In step 1, patients ventilated with VCV as per the ARDSnet protocol were subjected to EIT analysis. In step 2, patients were switched to APRV. Ventilation parameters, arterial blood gas analysis and percentage of tidal ventilation distribution in the four ROIs were recorded at steps 1 and 2. Analyses were performed by paired t test.

## Results

**Figure 1:**
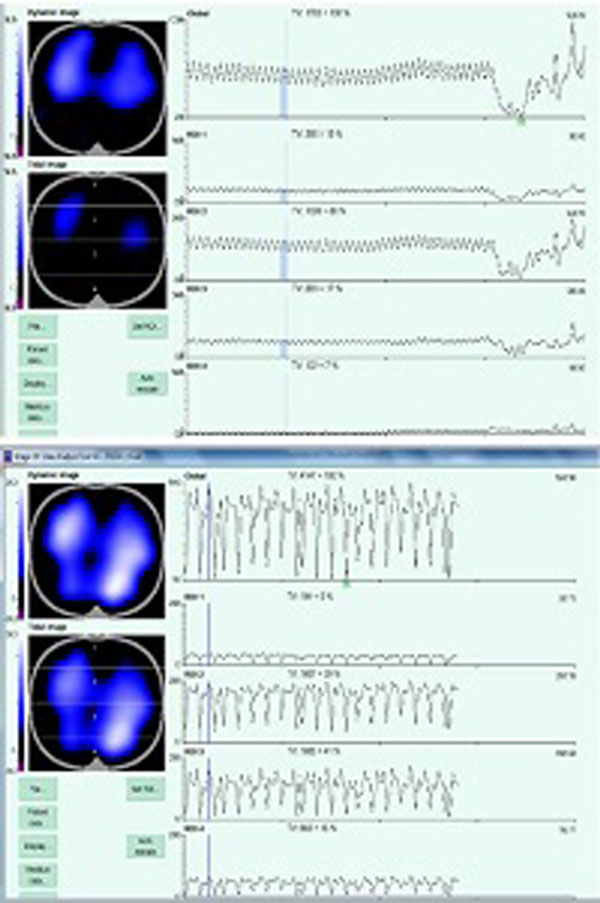
**EIT comparison of VCV and APRV**.

Patients on VCV had P/F ratio of 79.5 ± 12.5 with PEEP of 14.16 ± 1.32 There was a significant improvement in P/F ratios on switching to APRV (126.16 ± 23.69, *P *= 0.002) at 30 minutes of ventilation on APRV. There was a trend to decrease in FiO_2 _(0.82 ± 0.15 vs. 0.68 ± 0.10, *P *= 0.068) and PCO_2 _(52.5 ± 6.15 vs. 45.00 ± 8.67, *P *= 0.071) and increase in PaO_2 _(65.83 ± 14.53 vs. 84.83 ± 12.22, *P *= 0.056) at step 2. The proportional distribution of ventilation in the dorsal ROI 3 and ROI 4 also improved on switching to APRV. TV in ROI 3 during VCV, 12.76 ± 6.76%, improved to 24.58 ± 6.61% (*P *= 0.067). Similarly TV in ROI 4 during VCV, 24.58 ± 6.61%, improved to 26.6 ± 6.09% (*P *= 0.068). Due to small sample size, improvement in TV in dorsal ROIs was not statistically significant. Upper panel of Figure 1 shows end-inspiratory and end-expiratory images of EIT on VCV with poor TV in dorsal ROI 3 and ROI 4. Lower panel of figure shows two EIT images at Phigh = 30 cm showing improved TV in dorsal ROIs.

## Conclusion

EIT may help to identify patients with severe ARDS on VCV with a potential of increasing recruitment by tidal redistribution of ventilation with APRV.

